# Latent tuberculosis infection (LTBI) in health-care workers: a cross-sectional study at a northern Peruvian hospital

**DOI:** 10.3389/fmed.2023.1295299

**Published:** 2023-11-30

**Authors:** Edinson Dante Meregildo-Rodriguez, Verónica Yuptón-Chávez, Martha Genara Asmat-Rubio, Gustavo Adolfo Vásquez-Tirado

**Affiliations:** ^1^Escuela de Medicina, Universidad César Vallejo, Trujillo, Peru; ^2^Servicio de Laboratorio Clínico, Hospital Regional Lambayeque, Chiclayo, Peru; ^3^Escuela de Posgrado, Universidad Privada Antenor Orrego, Trujillo, Peru; ^4^Escuela de Medicina, Universidad Privada Antenor Orrego, Trujillo, Peru

**Keywords:** tuberculosis, latent tuberculosis, health personnel, interferon-gamma release tests, infection control

## Abstract

**Background:**

Healthcare workers (HCWs) have a higher risk of latent tuberculosis infection (LTBI) and active tuberculosis than the general population. In HCWs, the risk of tuberculosis infection depends on the local tuberculosis prevalence, HCWs’ characteristics, the healthcare facility, and prevention and control measures. We aimed to estimate the prevalence and risk factors for LTBI in HCWs at a northern Peruvian hospital.

**Methods:**

This study had two phases: (1) a cross-sectional phase involving recruitment, history taking, and sampling for the Interferon-Gamma Release Assays (IGRA test), and (2) a prospective follow-up of IGRA-positive participants. We enrolled direct and non-direct patient caregivers among HCWs. We defined an LTBI case if the IGRA test was positive and clinical, laboratory, and radiological evaluations for active tuberculosis were negative.

**Results:**

We recruited 308 participants between November 2022 and May 2023. The mean age was 38.6 ± 8.3 years. Over 75% of the participants were female. The most common job category was technicians (30.5%), physicians (22.7%), nurses (20.5%), and other HCWs groups (17.5%). Most participants worked in hospital wards (28.2%), diagnostics departments (16.9%), and critical care departments (15.6%). The LTBI prevalence among HCWs was 17.86% (95% CI 13.84–22.70). In multivariate analysis, after adjusting for age, time working in our hospital, and family history of tuberculosis, males had a higher risk of LTBI (aPR 1.69, 95% CI 1.01–2.77) than females. Working for more than 10 years increased the risk of LBTI (aPR 2.4, 95% CI 1.44–3.97) compared to working for ≤10 years. Even further, participants who had worked for more than 20 years had an aPR of 4.31 (95% CI 1.09–13.65) compared to those with ≤10 years. Similarly, occupational exposure increased the risk of LTBI (aPR 2.21, 95% CI 1.27–4.08) compared to those HCWs not occupationally exposed.

**Conclusion:**

The LTBI prevalence in HCWs at a northern Peruvian hospital was lower compared to other Peruvian cities. Males, more experienced, and occupational exposed HCWs are at higher risk of LTBI. LTBI prevalence in Peruvian HCWs is still high. More studies are needed to address some aspects this study has not examined.

## Introduction

Tuberculosis is the most widespread infectious disease (1, 2). Globally, tuberculosis remains one of the top 10 causes of death and the leading cause of a single infectious agent disease, even ahead of human immunodeficiency virus (HIV) infection (1–3). In Peru, tuberculosis is endemic, ranking as the fifteenth cause of death and predominantly affecting the poorest population (4). According to the Pan-American Health Organization, Peru has the second-highest tuberculosis burden in the Latin American and Caribbean (5).

Latent tuberculosis infection (LTBI) is a persistent immune response to stimulation by *Mycobacterium tuberculosis* (MTB) antigens without evidence of active disease (6). Approximately 25–30% of the world’s population has LTBI. About 10% of LTBI cases, especially during the first 2 years, may progress to active tuberculosis (7). Several factors could increase the risk of LTBI reactivation. For example, HIV infection, hemodialysis, immunosuppressive therapy, malignancy, diabetes mellitus, and more (6–8).

Healthcare workers (HCWs), due to their sustained occupational exposure to MTB, face a higher risk of LTBI and active tuberculosis (9–11). Tuberculosis constitutes a critical occupational hazard for HCWs, especially in areas with a high disease burden. The situation is more alarming in healthcare centers with limited infection prevention and control measures (11–13).

In developing countries, the prevalence of LTBI in HCWs is 54% (range 33–79%) (14). Furthermore, studies have shown a high LTBI prevalence in health sciences students attending primary care centers and specialized pulmonary hospitals (15–19). The risk of LTBI in HCWs depends on the local prevalence of tuberculosis, the characteristics of the healthcare facility and the HCWs’ activities, and the effectiveness of prevention and control measures (12, 13, 20, 21).

Tuberculosis is an occupational and notifiable disease in Peru (4, 22). In recent years, the incidence of tuberculosis among HCWs has decreased. This apparent reduction is likely due to an improved reporting system, which helps avoid duplication of notifications (4). Among tuberculosis cases among healthcare personnel in Peru, 57% corresponded to the public health system, 36% to the private social security system, and 7% to other institutions (4, 22).

The most important cornerstones for the prevention and control of tuberculosis are early detection of cases, diagnosis, and appropriate treatment (23). Addressing LTBI through screening and TB preventive treatment (TPT) is critical to ending the TB epidemic by 2035 (24). Since the risk of progression of LTBI into active tuberculosis is notably higher in recent converters (7), this is why TPT is indicated in this group (25). However, the costs to scale up LTBI screening and TPT programs are prohibitive for many national TB programs in resource-limited countries such as Peru, which are already struggling to provide active TB screening and treatment (24). Indeed, in Peru, the Tuberculosis Control Program does not contemplate an active search for LTBI cases, not even in the highest-risk populations such as HCWs (14, 17, 26). Therefore, we aimed to estimate the prevalence and risk factors for LTBI among HCWs at the “Hospital Regional Lambayeque” (HRL). HRL is a 500-bed, high-resolution health center in northern Peru. The results from this study contribute to a better understanding of the epidemiology of tuberculosis in one of the populations at the most significant tuberculosis risk, namely HCWs. This information will help plan public policies aimed at protecting the HCWs. Furthermore, this could serve as a foundation for future studies.

## Materials and methods

### Population

We use the term “healthcare workers” (HCWs) to refer to anyone working in the healthcare field, including direct and non-direct patient caregivers.

### Sample size

Based on data reported by Soto-Cabezas et al. in a similar Peruvian study (14), an expected prevalence of 60%, a 95% confidence interval (95% CI), and a design effect 1, we calculated a sample size of 298 participants using EPIDAT 4.2 (27). Initially, we intended to conduct a stratified random sampling considering the total number of hospital workers (approximately 1,800) stratified stratified into seven occupational groups: (1) physicians, (2) nurses, (3) technicians, (4) pharmacists, (5) other HCWs (obstetricians, nutritionists, psychologists, medical technologists, and biologists), (6) administrative support staff (secretaries, administrators, statisticians, and engineers), and (7) housekeeping personnel (cleaning, laundry, and disinfection staff).

### Study design

This study had two phases: (1) a cross-sectional phase (including participants recruiting and sample collection for the IGRA test), which every participant completed in a single day, and (2) a prospective follow-up of IGRA-positive participants between November 1, 2022, and May 31, 2023. Our study included HCWs from various occupational groups, as we detailed above. We collected information on sociodemographic determinants and possible factors associated with LTBI. The inclusion criteria were: (1) HCWs who freely and voluntarily agreed to participate and (2) HCWs aged 18 years or older. The exclusion criteria were: (1) participants with a history of previous tuberculosis disease or those likely to produce false negative results in the IGRA test (28, 29), such as anergic or immunocompromised patients (e.g., those with decompensated diabetes mellitus, cirrhosis, chronic kidney disease, cancer, HIV infection, etc.).

### Variables

The dependent variable was LTBI. Independent variables were age, sex, place of residence (district and province), profession or occupational group, working area, previous disease (comorbidity), family history of tuberculosis, occupational exposure to tuberculosis, time spent in the hospital (Time 1), and total time spent working as an HCWs (Time 2).

### Definitions

#### LTBI

There is no universal definition or gold standard test for LTBI (30). Most published studies endorsed the definition of LTBI by the CDC (31). Then, in this study, following the CDC recommendations (10, 26, 28, 29, 31, 32), if a participant tested positive on IGRA, clinical evaluation, at least two sputum samples to investigate acid-fast bacilli (AFB), and a chest X-ray (CXR) was performed to rule out active disease. Neither the CDC nor the WHO have endorsed including sputum culture or molecular methods for diagnosing LTBI (30, 31).

#### Healthcare worker (HCW)

We considered the following groups of HCWs: physicians (assistants, residents, and general practitioners), nurses, technicians, pharmacists, other HCWs (obstetricians, nutritionists, psychologists, medical technologists, and biologists), administrative staff, (secretaries, administrators, statisticians, and engineers), and housekeeping personnel (cleaning, laundry, and disinfection staff). *History of tuberculosis*: if a participant had a history of tuberculosis at least once. *Working area*: department, area, or service where participants work. *Service time*: the number of years of service in the health facility. *Comorbidity*: any chronic condition that may predispose to complications or cause functional impairment. *Direct patient caregivers*: This category includes physicians, nurses, and technicians, among others. *Non-direct patient caregivers:* administrative, cleaning, and maintenance personnel.

### Recruitment

After random sampling, we invited HCWs to participate using an informed consent form. If the HCWs agreed to participate, the researcher team reviewed their personal information to ensure they met the inclusion criteria and did not meet the exclusion criteria. Subsequently, the research staff collected data on demographic characteristics and other variables of interest.

### Sample collection and processing

In cases where participants accepted and provided authorization, they were sent to the HRL laboratory for the IGRA test. Trained laboratory personnel collected a 5 ml blood aliquot from each participant. Blood samples were processed using a CO_2_ incubator without recharging, stereoscope, and Auto-Pure 20B equipment. Of the two IGRA tests approved by the US Food and Drug Administration (FDA) available in most countries, we used the T–SPOT^®^ TB test (Oxford Inmunotec^®^) for sample processing. Samples were processed following the guidelines outlined in the Peruvian biosafety manuals from the National Institute of Health, which govern the sample collection process, handling, and disposal of biological waste products.

### Follow-up

The participants who tested positive in the IGRA test were followed up via telephone calls at 2-week intervals, looking for any symptoms that suggested active tuberculosis disease. Our protocol stated performing CXRs and at least two sputum samples for AFB for all IGRA-positive participants. If judged necessary by the researchers, HCWs with LTBI underwent further evaluation by an internist, pulmonologist, or infectious disease specialist.

### Data collection and analysis

We created a database using Excel^®^ and subsequently exported this data to R^®^ 4.2.226 software for analysis. We calculated absolute and relative frequencies, central tendency measures, and dispersion measures. We estimated the prevalence of LTBI using the total number of IGRA-positive HCWs as the numerator and the number of HCWs included in the IGRA survey as the denominator. Independent variables were stratified based on LTBI status.

To identify the independent variables that best predict LTBI, we conducted both bivariate and multivariate analyses using Poisson regression with robust variance (utilizing the *glm* function from quasipoisson regression family models). In the bivariate analysis, we employed the Chi-square (χ^2^) or Fisher’s exact test to explore associations between categorical variables. In the final regression models, we included variables with a *p*-value of ≤ 0.20 from the bivariate analysis. Crude and adjusted prevalence ratios (PR) and their respective 95% confidence intervals (95% CI) were calculated. A *p*-value ≤ 0.05 was considered statistically significant.

To assess the linearity assumption in the regression model, we utilized the Koenker-Bassett test. If the result of this test was not statistically significant (*p* > 0.05), we considered the assumption fulfilled (33, 34). Three multivariate regression models were constructed. Akaike information criterion (AIC) values and Area Under the Curve (AUC) of Receiver Operating Characteristics (ROC) were calculated for each model to compare and select the best one. The AIC measures how well a model fits the data, with lower AIC values indicating a better fit (35, 36). The AUC is a measure of a model’s ability to discriminate between positive and negative cases. A higher AUC indicates that the model better differentiates between the groups (37–39). The assumption of no multicollinearity was tested using the variance inflation factor (VIF). VIF values <6 indicated no multicollinearity, while VIF > 10 indicated significant multicollinearity (33).

### Ethical aspects

The HRL and Cesar Vallejo University Research Ethics Committees approved the protocol with the codes 0922-047-21CEI and 015-CE-FCS-UCV-21, respectively.

This article was reported following the guidelines of the STROBE Statement for cross-sectional studies (40) (Supplementary Table 3).

## Results

### Clinical-epidemiological characteristics

We recruited 308 HCWs between November 2022 and May 2023 (Table 1). The mean age was 38.6 ± 8.3 years (22 to 67 years). Seventy-six percent of the participants were female. Furthermore, more than 88% of participants resided in the province of Chiclayo. At the beginning of the study, our hospital had about 1,800 workers. Of these, approximately half were direct patient-caregiver workers. The initial protocol contemplated carrying out a stratified sampling according to occupational groups. However, this was not possible since the study considered the free participation of HCWs. Several HCWs refused to participate, which left several occupational groups underrepresented. This fact also skewed the distribution of some variables, such as age, with most study subjects being young. Consequently, our randomization process was incomplete.

**TABLE 1:** Clinical-epidemiological characteristics of the HCWs.

Variable	*N* = 308	%
**Age (years) mean (SD)**	38.53 (8.26)	**–**
<40	191	62.0
40–60	111	36.0
>60	6	1.9
**Sex**		
Female	234	76.0
Male	74	24.0
**Province**		
Chiclayo	272	88.3
Lambayeque	23	7.5
Ferreñafe	9	2.9
Others	4	1.3
**Occupation/profession**		
Physician	70	22.7
Nurse	63	20.5
Pharmacist	21	6.8
Healthcare technician	94	30.5
Other HC profesional*	33	10.7
Administrative support staff	17	5.5
Housekeeping staff	10	3.2
**Department or working area**		
Diagnostic support department	52	16.9
Hospital wards	87	28.2
Critical care department (ER and ICU)	48	15.6
Treatment support department	39	12.7
Outpatient care	36	11.7
Operating room and procedure areas	28	9.1
Administrative and support services	18	5.8
**Time 1 (years) mean (SD)**	4.97 (4.05)	**–**
0–10	262	85.1
>10–20	46	14.9
>20	0	0.0
**Time 2 (years) mean (SD)**	7.53 (5.19)	**–**
0–10	223	72.4
>10–20	79	25.6
>20	6	1.9
**Previous disease (comorbidity)**		
No	264	85.7
Yes	44	14.3
**Family history of tuberculosis**		
No	261	84.7
Yes	47	15.3
**Occupational exposure**		
No	117	38.0
Yes	191	62.0
**Outcome**		
No	253	82.1
Yes	55	17.9

*Other HC profesional* (obstetrician, nutritionist, psychologist, medical technologist, and biologist).

The most prevalent job category were technicians (30.5%), physicians (22.7%), and nurses (20.5%). Other HCWs groups, such as obstetricians, nutritionists, psychologists, medical technologists, and biologists, accounted for 10.7% of the HCWs. Administrative support staff, such as secretaries, administrators, statisticians, and engineers (5.5%), and housekeeping personnel, such as cleaning, laundry, and disinfection staff (3.2%), represented a minority of the participants. Most participants worked in surgical or clinical hospital wards (28.2%), the diagnostic support departments (including laboratory, pathology, and imaging services) (16.9%), the critical care departments (ER and ICU areas) (15.6%), the treatment support department (pharmacy) (12.7%), outpatient care areas (clinics and occupational health areas) (11.7%), operating room and procedure areas (9.1%), and administrative and support services (5.8%). It is noteworthy that, in Peru, medical technologists (MTs) are professionals with different specialties and work in other areas, e.g., laboratory, pathology, radiology, and rehabilitation. Due to their occupation, such as handling biological samples, these professionals have a higher risk of exposure than other healthcare workers. Indeed, in our study, 16 MTs participated, of which 15 belonged to the laboratory and 1 to pathology. Four of 16 MTs had LTBI.

We examined two exposure times: the duration a worker spent in our hospital (Time 1) and the total time a participant worked in any healthcare facility, including the HRL (Time 2). The mean Time 1 was 4.97 ± 4.05 years. More than 85% of participants had ten or fewer years of experience in our hospital, and no one had spent more than 20 years there. The mean Time 2 was 7.53 ± 5.19 years. Seventy-two percent of the participants had worked for ten or fewer years, while 25.6% had worked between 10 and 20 years. However, fewer than 2% of HCWs had worked for over 20 years.

About 85% of HCWs had no comorbidity or family history of tuberculosis (Table 1). Among the 44 HCWs who reported any comorbidity, 17 had hypertension, 10 had asthma/chronic obstructive pulmonary disease, 7 had pre-diabetes/diabetes, 7 had obesity, and 12 reported other comorbidities (Supplementary Table 1). In contrast, considering only those HCWs with LTBI, 62% reported a history of occupational exposure, i.e., contact with any college or patient with tuberculosis. Of these, 29.3% were healthcare technicians, 28.8% were physicians, 19.4% were nurses, and 16.2% were of other occupational groups. Pharmacists, administrative, and housekeeping personnel accounted for less than 10% (Supplementary Table 4).

### Prevalence of LTBI

We found that the prevalence of LTBI among HCWs was 17.86% (95% CI 13.84–22.70). Until the closure of this study (May 31, 2023), all patients with a positive IGRA test remained asymptomatic and had had a chest x-ray and at least three negative sputum smear tests. We did not perform sputum cultures or molecular tests on participants with a positive IGRA result. The follow-up period ranged between 14 and about 180 days.

### Risk factors for LTBI

In the bivariate analysis, we found that the following variables were associated with LTBI: age, sex (male), Time 1, Time 2, previous disease, family history of tuberculosis, and occupational exposure (Table 2). We explored three multivariate models. Model 1 included age, sex, Time 1, Time 2, previous disease, family history of tuberculosis, and occupational exposure; Model 2 encompassed age, sex, Time 2, prior disease, and occupational exposure; and Model 3 featured age, Time 2, previous disease, and occupational exposure (Table 3). Model 1 had a lower AIC and a higher AUC (Supplementary Table 2). These indicators suggest that Model 1 outperformed both Model 2 and Model 3 regarding goodness of fit and discriminant capacity.

**TABLE 2:** Bivariate analysis: factors associated with the risk of LTBI.

Variable	Outcome			
	LTBI (*N* = 55)	No-LTBI (*N* = 253)	Crude PR* (95% CI)	*P*
**Age (years)**	**–**	**–**	**0.97 (0.94–1.00)**	**0.073**
**Age range**				
<40	39 (20.4)	152 (79.6)	Reference	
40–60	15 (13.5)	96 (86.5)	0.66 (0.38–1.12)	0.137
>60	1 (16.7)	5 (83.3)	0.82 (0.07–3.37)	0.826
**Sex**				
Female	37 (15.8)	197 (84.2)	Reference	
Male	18 (24.3)	56 (75.7)	1.54 (0.91–2.54)	0.100
**Province**				
Chiclayo	54 (19.9)	218 (80.1)	Reference	
Lambayeque	1 (4.3)	22 (95.7)	2.190016e-01	0.091
Ferreñafe	0 (0.0)	9 (100.0)	1.540841e-07	0.988
Others	0 (0.0)	4 (100.0)	1.540841e-07	0.992
**Occupation/profession**				
Physician	12 (17.1)	58 (82.9)	Reference	
Nurse	11 (17.5)	52 (82.5)	1.018519e + 00	0.961
Pharmacist	2 (9.5)	19 (90.5)	5.555556e-01	0.392
Healthcare technician	23 (24.5)	71 (75.5)	1.427305e + 00	0.267
Other HC profesional*	5 (15.2)	28 (84.8)	8.838384e-01	0.796
Administrative support staff	2 (11.8)	15 (88.2)	6.862745e-01	0.584
Housekeeping staff	0 (0.0)	10 (100.0)	1.784430e-07	0.987
**Department or working area**				
Diagnostic support department	12 (23.1)	40 (76.9)	Reference	
Hospital wards	11 (12.6)	76 (87.4)	0.55 (0.26–1.17)	0.117
Critical care department (ER and ICU)	11 (22.9)	37 (77.1)	0.99 (0.46–2.11)	0.985
Treatment support department	7 (17.9)	32 (82.1)	0.78 (0.32–1.79)	0.565
Outpatient care	7 (19.4)	29 (80.6)	0.84 (0.34–1.94)	0.695
Operating room and procedure areas	4 (14.3)	24 (85.7)	0.62 (0.20–1.63)	0.366
Administrative and support services	3 (16.7)	15 (83.3)	0.72 (0.19–2.08)	0.583
**Time 1 (years)**			1.04 (0.98–1.10)	0.180
0–10	42 (16.0)	220 (84.0)	Reference	
>10–20	13 (28.3)	33 (71.7)	1.76 (0.97–3.03)	0.050
>20	0 (0.0)	0 (0.0)	–	–
**Time 2 (years)**			1.06 (1.02–1.09)	0.002
0–10	31 (13.9)	192 (86.1)	Reference	
>10–20	21 (26.6)	58 (73.4)	1.91 (1.14–3.15)	0.012
>20	3 (50.0)	3 (50.0)	3.60 (1.01–9.28)	0.021
**Previous disease (comorbidity)**				
No	53 (20.1)	211 (79.9)	Reference	
Yes	2 (4.5)	42 (95.5)	0.23 (0.05–0.66)	0.024
**Family history of tuberculosis**				
No	43 (16.5)	218 (83.5)	Reference	
Yes	12 (25.5)	35 (74.5)	1.55 (0.83–2.70)	0.141
**Occupational exposure**				
No	12 (10.3)	105 (89.7)	Reference	
Yes	43 (22.5)	148 (77.5)	2.20 (1.26–4.07)	0.008

*Other HC professionals* (midwife, nutritionist, psychologist, medical technologist, and biologist.

**TABLE 3:** Multivariate analysis: factors associated with the risk of LTBI.

Variable	Model 1		Model 2		Model 3	
	aPR (95% CI)	*p*	aPR (95% CI)	*p*	aPR (95% CI)	*p*
**Age (years)**						
<40	Ref.		Ref.		Ref.	
40–60	0.54 (0.30–0.92)	0.0296	0.51 (0.28–0.87)	0.01864	0.54 (0.30–0.92)	0.0294
>60	1.03 (0.09–4.50)	0.9745	1.11 (0.09–4.81)	0.90915	1.36 (0.11–5.73)	0.7412
**Sex**						
Female	Ref.		Ref.		–	
Male	1.69 (1.01–2.77)	0.0418	1.71 (1.01–2.82)	0.0396	–	–
**Time 1 (years)**						
0–10	Ref.		**–**		**–**	
>10–20	0.99 (0.47–2.08)	0.9842	–	–		
>20	–	–	–	–	–	–
**Time 2 (years)**						
0–10	Ref.		Ref.			
>10–20	2.33 (1.22–4.23)	0.0076	2.48 (1.47–4.11)	0.0006	2.41 (1.44–3.97)	0.0007
>20	4.31 (1.09–13.65)	0.0217	4.96 (1.38–13.31)	0.0396	4.75 (1.34–12.57)	0.0054
**Previous disease (comorbidity)**						
No	Ref.		Ref.		Ref.	
Yes	0.17 (0.037–0.51)	0.0070	0.18 (0.04–0.53)	0.0087	0.18 (0.03–0.54)	0.0010
**Family history of tuberculosis**						
No	Ref.		–	–	–	
Yes	1.59 (0.85–2.79)	0.1254	–	–	–	–
**Occupational exposure**						
No	Ref.		Ref.		Ref.	
Yes	2.28 (1.30–4.22)	0.006	2.16 (1.24–4.01)	0.0101	2.21 (1.27–4.08)	0.0076

aPR, adjusted prevalence ratio.

According to Model 1, after adjusting for age, Time 1, and family history of tuberculosis, the factors independently associated with the risk of LTBI among HCWs were sex, Time 2 and occupational exposure to tuberculosis. Our findings indicated that male HCWs had a PR of 1.69 (95% CI 1.01–2.77) compared to female HCWs. HCWs who had worked in the field for over 10 years had a PR of 2.33 (95% CI 1.22–4.23) compared to those with 10 or fewer years of experience. Similarly, HCWs who had worked for more than 20 years had a PR of 4.31 (95% CI 1.09–13.65) compared to those with less than 10 years of experience. Likewise, individuals with a history of occupational exposure had a PR of 2.28 (95% CI 1.30–4.22) compared to HCWs without occupational exposure. On the contrary, a history of previous illness (comorbidity) was not a risk factor for LTBI and was associated with a PR of 0.17 (95% CI 0.037–0.51).

## Discussion

### Clinical-epidemiological characteristics

This study included 308 healthcare workers who provided direct and non-direct patient care. It represents the most extensive investigation to detect LTBI in Peruvian HCWs using the T-SPOT^®^ IGRA method and the second such study when considering research employing the tuberculin skin test (TST).

Soto Cabezas et al. (14) conducted a descriptive study using a secondary database of HCWs from a sentinel tuberculosis surveillance program at a health facility in Lima, Peru. Their study involved 150 HCWs between March and June 2008, and they utilized the QFT-GIT^®^ IGRA method. Similarly, Escombe et al. (16) conducted a cohort study between 2005 and 2006 at a public hospital in Lima, Peru. They invited 70 direct and non-direct patient care ED staff and performed QFT-GIT^®^ IGRA testing at baseline and 1 year later.

Sedamano et al. (41) conducted a cross-sectional study in a high-burden tuberculosis setting of primary care health centers in Lima, Peru, between September 2014 and March 2015. They invited 240 HCWs but administered TST to only 190 of them. Similarly, Alonso-Echanove et al. (15) screened 1,600 HCWs, identifying 44 with presumptive active tuberculosis; however, only 270 HCWs underwent a TST.

Finally, Ju Wang et al. (42) conducted a retrospective study at a hospital in Lima, Peru, intending to identify demographic factors associated with LTBI. They reviewed the records of 1,278 HCWs who worked at the hospital between 2010 and 2013, applying the TST to only 871.

We found that most HCWs were relatively young; the mean age was 38.6 ± 8.3 years. This finding is consistent with other Peruvian studies on LTBI among HCWs, which found that most participants were in their thirties and forties (14, 15, 41). Two other publications, including Peruvian HCWs, did not report the participant’s ages (16, 42). Furthermore, two systematic reviews on tuberculosis infection in HCWs (43, 44), one specifically conducted on low- and middle-income countries (LMICs) (43), did not provide information on the participants’ ages.

In our study, more than three-quarters of the participants were females because more females than males agreed to participate. This finding is consistent with Soto Cabezas et al.’s (14) and Sedamano et al.’s (41) studies. In the former study, 77% of the participants were females, and in the last one, more than 80% were females. Alonso-Echanove et al. included similar numbers of male and female participants (15). However, the studies by Escombe et al. (16) and Ju Wang et al. (42) did not detail the participants’ sexes.

Our study incorporated a broad spectrum of HCWs, including direct patient caregivers (physicians, nurses, etc.), and non-direct patient caregivers (cleaning, logistic support, and administrative personnel). The most common occupation/profession categories were healthcare technicians, followed by physicians and nurses; administrative and housekeeping personnel comprised less than 10% of the participants. Therefore, our findings are consistent with other studies. Soto Cabezas et al. (14) found that most participants were clinical staff, administrative and paramedical personnel, nurses, and obstetricians. Sedamano et al. (41) also reported that most participants were clinical staff, followed by administrative and paramedical staff. In both studies, nursing technicians outnumbered nurses, obstetricians, and physicians. Indeed, in both studies, physicians accounted for less than 10% of the HCWs (14, 41). On the contrary, Alonso-Echanove et al. invited only “direct patient caregivers” working in medicine wards, the ED, ICU, or laboratory and included more physicians than other groups (15). Escombe et al. also collected direct patient caregivers and support personnel, such as administrative, cleaning, and security staff; however, they all worked in the ED (16). The paper by Ju Wang et al. (42) has only been published in abstract form; therefore, it is impossible to ascertain the sociodemographic characteristics of this population.

Regarding the working area, we found that most participants worked in hospital wards, followed by diagnostic support areas, critical care areas, pharmacies, outpatient clinics areas, and administrative and support services. Among the studies conducted in Peru, the only one that reported the location or working area was Alonso-Echanove et al. (15) They described that most HCWs worked in the central laboratory, followed by the medicine wards and the ED/ICU. However, it is essential to clarify that their research aims were quite different from the other studies performed in Peru (14, 16, 41, 42). Alonso-Echanove et al. examined HCWs with presumptive active tuberculosis and assessed the risk factors for occupational transmission (15). In contrast, Soto Cabezas et al. (14), Sedamano et al. (41), and Ju Wang et al. (42) did not mention the working location of the HCWs. Additionally, all the HCWs included in Escombe et al.’s study (16) worked in the ED.

We found that the participants had spent an average of less than 5 years laboring in our hospital. Indeed, almost 90% of participants had less than 10 years of working in the HRL, and no one had worked for more than 20 years. Similarly, the participants had been laboring as HCWs for an average of less than 8 years. Seventy-two percent of the participants had labored ten or fewer years as HCWs; a quarter worked between 10 and 20 years, but fewer than 2% of the HCWs had worked for more than 20 years. These findings concord with Sedamano et al. (41), who stated that the median time working in the health center was 4 years, and working as a HCW was 10 years. Similarly, Soto Cabezas et al. (14) found that 28.6% of the participants worked as HCWs for less than 5 years, 16.5% between six and 10 years, and 54.9% had worked more than 10 years. Conversely, Alonso-Echanove et al. (15) only reported that 102 out of 156 HCWs working in clinical areas for at least 1 year were TST positive, and 37 out of 52 HCWs working in laboratory areas for at least 1 year were TST positive. Contrarily, Escombe et al. (16) analyzed the hours worked in the ED, outside the ED, and the total hours worked by the clinical and non-clinical HCWs. The paper by Ju Wang et al. (42) did not provide enough detail in this regard.

In this study, more than 85% of HCWs were not comorbid. This finding is likely explained by the fact that most participants were relatively young. Soto Cabezas et al. (14) did not mention the comorbidities of the HCWs included. Alonso-Echanove et al. (15) described that 6% of HCWs with confirmed tuberculosis had comorbidities. Similarly, Sedamano et al. (41) found that 28.75% of the participants had any comorbidity. But, Escombe et al. and Ju Wang did not specify the comorbid conditions of their participants (16, 42).

Only 15.3% of our participants had a family member with a history of tuberculosis. In the study by Soto Cabezas et al. (14), 36.7% of the HCWs had a history of contact with family or friends with tuberculosis. In the same way, Sedamano et al. stated that 30.5% of their participants had a tuberculosis household contact (41). Escombe et al., Alonso-Echanove et al. and Ju Wang et al. did not report this antecedent (15, 16, 42).

We found that 62% of our participants reported a history of occupational exposure, i.e., contact with any HCW or patient with known or suspected tuberculosis. This percentage is lower than that reported by Sedamano et al. (41) and Soto Cabezas et al. (14), who indicated that 85.4 and 81.3% of their participants had ever directly cared for or treated patients with tuberculosis in their career, respectively. On the other hand, Alonso-Echanove et al. (15) did not state the proportion of their HCWs with this history; however, they described that 57 out of 71 HCWs helping in sputum collection were TST-positive, and 106 out of 142 HCWs who had contact with a person with active tuberculosis were TST-positive. In contrast, Escombe et al. and Ju Wand et al. did not detail the occupational exposure of the HCWs included (16, 42).

### Prevalence of LTBI

We found a prevalence of LTBI among HCWs of 17.86% (95% CI 13.84–22.70). This prevalence is lower than those reported by other authors in Peru (14–16). In the study by Soto Cabezas et al. (14), the LTBI prevalence, based on the QFT-GIT IGRA method, was 56.0% (95% CI 46.7–63.4%). In the assay conducted by Escombe et al. (16), the prevalence at baseline was 55.7% according to the QFT-GIT IGRA method. Alonso-Echanove et al. (15) reported that 36 HCWs had confirmed pulmonary tuberculosis. Of 270 HCWs who underwent a TST, 170 (63.9%) had a TST-positive reaction. Similarly, Sedamano et al. (41) found that, excluding those participants who did not attend the TST reading and those with a previous TST, the prevalence of LTBI was 56.5% (95% CI: 49.22–63.55%). Also, Ju Wang et al., using TST, noted a prevalence of LTBI of 45.7% in HCWs.

We must note that this is the first study on the prevalence of LBTI in a department in northern Peru. All other previously mentioned studies were conducted in Lima, Peru (14–16, 41, 42). Although there are no previous reports on the prevalence of LTBI in HCWs from departments other than Lima, there are studies on the prevalence of tuberculosis in the general population. Therefore, the main reason for these differences in the prevalence of LTBI found in our study and other research performed in Lima, Peru, is due to different tuberculosis infection rates in the general population and HCWs among the various Peruvian localities (4, 45, 46).

The prevalence of tuberculosis in Lima and other departments of Peru is high. The Peruvian Ministry of Health reported 13,262 new tuberculosis cases in Peru in 2022, of which 5,563 were in Lima, representing an incidence rate of 12.1 cases per 100,000 inhabitants nationwide. The department with the highest prevalence of tuberculosis in Peru is Lima, with 17.3 cases per 100,000 inhabitants, followed by some departments of southern Peru, such as Cusco 15.7 cases per 100,000 inhabitants, Puno 14.1 cases per 100,000 inhabitants, and Arequipa 13.2 cases per 100,000 inhabitants. The prevalence of tuberculosis in Lambayeque is 12.2 cases per 100,000 inhabitants. Indeed, Lima is classified as a region of “extremely high risk of tuberculosis”; meanwhile, the northern departments of Peru, such as Ancash, La Libertad, and Lambayeque, are considered regions of “low to moderate risk” (45–47).

Another possible reason for the discordance in the LTBI prevalence is the different rates of family history of tuberculosis and occupational exposure to tuberculosis, which were lower than those reported in other Peruvian studies. It is unlikely that other factors, such as the populations of HCWs, the settings of the health facilities, the study design, or the methods used to diagnose LTBI (TST vs. QFT-GIT) accounted for these disparities in prevalence since the prevalence reported in Lima were quite similar.

Two systematic reviews have explored the prevalence of LTBI in HCWs in other countries. Apriani et al. in 2019 conducted a systematic review of 85 studies from 26 LMICs. They found an LTBI prevalence of 14–98% (mean 49%) based on TST and 9–86% (mean 39%) based on IGRA. As expected, the countries with the highest tuberculosis incidence in the general population had the highest LTBI prevalence in HCWs (TST pooled estimate 55, 95% CI 41–69% and IGRA pooled estimate 56, 95% CI 39–73%) (43).

Accordingly, in 2021, Lee et al. published a systematic review based on 39 studies from America, Asia, Europe, and Africa, reporting that the global burden of LTBI in the general population was 23.0% (95% CI 20.4–26.4%) in 2014. In contrast, the LTBI prevalence in HCWs based on TST was 29.94%. In South America, the reported LTBI prevalence among HCWs varied from 56% in Peru, 39.4% in Brazil, 15.4% in Cuba, 5.7% in Canada, and 3–4.2% in the USA (34).

### Factors associated with LTBI

In the bivariate analysis, we found that the following variables were associated with LTBI: age, sex (male), the time spent working as an HCW at our hospital (Time 1), the total time spent working as an HCW (Time 2), having a previous disease or family history of tuberculosis, and occupational exposure to tuberculosis (Table 2).

Our results showed that sex, Time 2, and occupational exposure to tuberculosis were independently linked with the probability of LTBI among HCWs in multivariate analysis, after controlling for age, Time 1, and family history of tuberculosis. Compared to women, men had an adjusted prevalence ratio (aPR) of 1.69 (95% CI 1.01–2.77). When comparing HCWs with more than 10 years of experience to those with 10 or fewer years, the aPR was 2.33 (95% CI: 1.22–4.23). In a similar vein, HCWs with more than 20 years of experience outperformed those with less than 10 years of experience with an aPR of 4.31 (95% CI 1.09–13.65). Additionally, when compared to HCWs without a history of occupational exposure, those with a previous occupational exposure had an aPR of 2.28 (95% CI 1.30 4.22).

Our findings are concordant with other studies conducted in Peru. Soto Cabezas et al. (14) reported that, in their final multivariate model, the only factor associated with LTBI was the service time, which is equivalent to Time 2 in our study. Indeed, participants with more than 10 years of service had an odds ratio (OR) of 2.3 compared to those with fewer than 5 years after adjusting for sex, place of birth, and contact with tuberculosis patients in their workplace. However, the authors noted the main limitation of their study was the use of secondary data, which restricted their ability to investigate other risk factors not included in their database. They also faced challenges related to a small sample size and incomplete data for some variables.

Similarly, Sedamano et al. (41), in their multivariate analysis, found that the only factor associated with TST positive result, after adjusting for overweight, household TB contact, and not using an N95 mask, was working as an HCW for more than 10 years (PR 1.52). The authors did not include age and the duration of working in the health center in their analysis since these factors are closely related to time spent working as an HCW, which is a more specific measure of tuberculosis exposure. This rationale is concordant with our results.

Alonso-Echanove et al. (15) assessed the risk factors for tuberculosis infection or disease in direct patient caregivers working in a third-level hospital in Lima, Peru. In multivariate analyses, they found that among clinical HCWs —those working in medicine wards, the ED, and the ICU) —having contact with a person with active tuberculosis (OR 9.62), assisting patients with sputum collection (OR 3.13), and the duration of time worked in the hospital (OR 1.0013)—which is equivalent in our study to Time 1—independently increased the risk of tuberculosis infection. Among laboratory HCWs, only using common staff areas remained significantly associated with an increased risk for tuberculosis infection (OR 16.44).

Escombe et al. (18), in a cohort study, aimed to investigate the annual risk of occupational tuberculosis infection among ED staff at a hospital in a low-resource, high-prevalence setting in Lima, Peru. They reported baseline and 1-year IGRA status, the annual incidence of tuberculosis infection, and infection control measures in HCWs. However, they did not perform a multivariate analysis to assess the factors related to the risk of tuberculosis infection. Furthermore, the authors noted that the sampling of the patients was incomplete and non-random, and not all patients were recruited.

Ju Wang et al. (42) reported that the demographic factors associated with LTIB included age, female sex, time worked in the hospital, and previous contact with a college with active tuberculosis. Individuals with latent tuberculosis infection (LTBI) tended to be older, more likely to be female, and had worked longer tenures in hospitals compared to those without LTBI. Individuals with a history of contact with a colleague with active tuberculosis were also more likely to have LTBI. However, the authors solely conducted a bivariate analysis, and they did not perform a multivariate analysis. Consequently, the results of this study could be biased or inaccurate. Unlike multivariate analysis, bivariate analysis does not allow for the control of other variables’ influence on the relationship between exposure and outcome variables. Multivariate analysis helps ensure more accurate results that better represents reality (48, 49).

According to our findings, comorbidity is not a risk factor for LTBI. We hypothesize that three reasons might explain this finding. First, except for seven patients with pre-diabetes or diabetes, most HCWs did not have comorbidities associated with a higher risk of tuberculous infection. Second, it is likely that these workers, aware of having comorbidities, take extreme precautionary measures. Third, the most critical determinants of tuberculous infection are the closeness of contact and infectiousness of the source. Conversely, the likelihood of developing active tuberculosis depends upon the intensity and duration of exposure and immunosuppressive conditions. Individuals with intense exposure are at greater risk of infection and disease development (50, 51).

A systematic review of the prevalence and incidence of LTBI in HCWs in LMICs, published in 2019 (43), concluded that the prevalence and incidence of a positive IGRA test were associated with years of work, work location, tuberculosis contact, and job category. The authors recognized some limitations: (1) the absence of a gold standard for diagnosing LTBI, (2) no guarantee that prevalence and incidence estimates of LTBI were accurate, and (3) they found substantial heterogeneity.

Regarding sex as a risk factor for tuberculosis in HCWs, studies have not been consistent. Soto Cabezas et al. (14) did not find a statistically significant association between sex and the risk of LTBI (aOR 2.2, 95% CI 0.9–5.7). Sedamano et al. and Alonso Echanove et al. reported that they included the variable sex in their bivariate analysis; however, this variable was not related to LTBI (15, 41). Consequently, sex was not included in the final model. On the contrary, Escombe et al. did not report information regarding the variable sex. Ju Wang et al. (42) stated that females had a higher risk of LTBI than males (54.9 vs. 45.1, *p* < 0.002). However, they did not perform a multivariate analysis. Similarly, in the systematic reviews by Apriani et al. (43) and Lee (44) et al., sex was not reported to be a risk condition for the incidence or prevalence of LTBI.

Our study has some limitations. First, this study was conducted in a single center. Second, many workers refused to participate, then a complete stratified sampling was impossible. Then, our randomization process was incomplete. Third, we did not explore other variables, such as infection control measures and the length of occupational exposure. Fourth, we noted a significant turnover of staff, e.g., from ED to hospitalization or ICU, or vice versa, which could have influenced LTBI rates. Fifth, we took an IGRA test only once.

Furthermore, due to the scarcity of research staff, it took us about 6 months to complete the application and reading of the IGRA test. This “delay” in the execution of the research may have influenced the prevalence of LTBI. As shown in two Peruvian studies, HCWs who were initially negative in the IGRA test may subsequently become positive or vice versa. In the study by Escombe et al. (16), eight of the 31 initial IGRA-negative participants became IGRA-positive 12 months later. Besides, of the 39 initial IGRA-positive participants, one became negative 18 months later. Similarly, in the study conducted by Bonifacio et al. (17), five of the 35 physicians initially negative in TST, retested after 1 year had converted, and one of these five also had active pleural TB.

Despite these limitations, the results of this study are relevant since this is the most extensive primary study conducted in Peru. In this study, the diagnosis of LTBI was established using the QFT-GIT technique based on a cell-mediated response. The result is not biased by previous BCG vaccination nor atypical mycobacterial infections. Indeed, evidence shows that QFT-GIT has a higher specificity than TST, especially in recent contacts with smear-positive patients (52–55).

## Conclusion

The prevalence of LTBI in HCWs from a northern Peruvian hospital is 18%. This rate is lower than that reported in other Peruvian studies. Male HCWs with more experience and occupational exposure history are at higher risk of LTBI. HCWs in LMICs with high- tuberculosis burden, such as Peru, still have a high prevalence of LTBI. More studies are needed to address the following: (1) the prevalence of LTBI in HCWs in other locations, (2) aspects of LTBI in HCWs that have not yet been examined, such as the role of genetics and environmental factors, and (3) interventions that could be used to reduce the prevalence of LTBI in HCWs.

## Data availability statement

The raw data supporting the conclusions of this article will be made available by the authors, without undue reservation.

## Ethics statement

The studies involving humans were approved by the Hospital Regional Lambayeque and Cesar Vallejo University Research Ethics Committees. The studies were conducted in accordance with the local legislation and institutional requirements. The participants provided their written informed consent to participate in this study.

## Author contributions

EM-R: Conceptualization, Data curation, Formal analysis, Funding acquisition, Investigation, Methodology, Project administration, Resources, Software, Supervision, Validation, Visualization, Writing – original draft, Writing – review and editing. VY-C: Conceptualization, Investigation, Methodology, Resources, Supervision, Validation, Writing – original draft, Writing – review and editing. MA-R: Investigation, Methodology, Writing – original draft, Writing – review and editing. GV-T: Investigation, Software, Writing – original draft, Writing – review and editing.
